# Two-Dimensional Angle Estimation of Two-Parallel Nested Arrays Based on Sparse Bayesian Estimation

**DOI:** 10.3390/s18103553

**Published:** 2018-10-19

**Authors:** Lu Chen, Daping Bi, Jifei Pan

**Affiliations:** Electronic Countermeasures College, National University of Defense Technology, Hefei 230037, China; bdpeei@163.com (D.B.); pjfeei@126.com (J.P.)

**Keywords:** decoupled estimation, sparse arrays, direction of arrival estimation, sparse Bayesian learning, degrees of freedom

## Abstract

To increase the number of estimable signal sources, two-parallel nested arrays are proposed, which consist of two subarrays with M sensors, and can estimate the two-dimensional (2-D) direction of arrival (DOA) of $M^{2}$ signal sources. To solve the problem of direction finding with two-parallel nested arrays, a 2-D DOA estimation algorithm based on sparse Bayesian estimation is proposed. Through a vectorization matrix, smoothing reconstruction matrix and singular value decomposition (SVD), the algorithm reduces the size of the sparse dictionary and data noise. A sparse Bayesian learning algorithm is used to estimate one dimension angle. By a joint covariance matrix, another dimension angle is estimated, and the estimated angles from two dimensions can be automatically paired. The simulation results show that the number of DOA signals that can be estimated by the proposed two-parallel nested arrays is much larger than the number of sensors. The proposed two-dimensional DOA estimation algorithm has excellent estimation performance.

## 1. Introduction

The DOA estimation of multiple signal sources plays an important role in fields such as radar, sonar, wireless communications and seismology. 2-D DOA estimation has important practical significance. Therefore, a large number of super-resolution DOA estimation algorithms have been proposed. One of the most popular methods is multiple signal classification (MUSIC) [1,2,3]. The MUSIC algorithm utilizes the orthogonality between signal subspace and noise subspace to achieve high-resolution estimation of signal angles [4,5,6,7,8]. This kind of angle estimation algorithm based on spatial orthogonality has some limitations [9,10,11,12]. That is to say, for a uniform array, whether it is a one-dimensional (1-D) array or a 2-D array, there is a problem that the number of physical elements M limits the number of signal sources K that can be estimated, namely, K<M [13].

In order to overcome this limitation, some nonuniform array structures have attracted wide attention. The nonuniform arrays can increase the degree of freedom (DOF) for signal estimation so that the number of signals that can be estimated is greater than the number of physical elements [14,15,16,17]. As a kind of nonuniform array, the minimum redundant array (MRA) was first proposed in Ref. [18]. This array structure can maximize the degree of freedom of parameter estimation when the number of elements is given. However, the disadvantage of this structure is that there is no closed expression for the position of elements. In Refs. [19,20], the generalized covariance matrix was proposed to enlarge the degree of freedom. However, the generalized covariance matrix is not positive semidefinite for a finite number of snapshots. In Ref. [21], nested arrays were proposed to estimate the DOAs of $O\left(M^{2}\right)$ signals with M elements. Due to the mutual coupling effect caused by the small sensor spacing of nested arrays, the coprime array structure was proposed in Ref. [22]. The element spacing of coprime arrays is sparse; nevertheless, the number of signal sources can be K≤MN with 2M+N−1 elements, where N denotes the sensor number of subarray 1, and 2M−1 denotes the sensor number of subarray 2. On the basis of one-dimensional sparse nonuniform arrays, Ref. [23] presented a novel nonuniform L-shaped array consisting of two-level nested arrays. A conjugate augmented spatial–temporal cross-correlation matrix (CAST–CCM) was constructed to estimate the two-dimensional angles. Another array structure used for 2-D angle estimation is the parallel array. Ref. [24] presents decoupled estimation for two parallel arrays (DETPA). By this method, the 2-D angle estimation problem was transformed into a 1-D search problem, which reduced the computational complexity of the original 2-D search algorithms. A two-parallel coprime array (TPCA) was proposed in Ref. [25]. By constructing a cross-correlation matrix of the received signals, 2-D angles were estimated using the sparse reconstruction technique. On the basis of two-parallel arrays and sparse reconstruction, Ref. [26] proposed to enhance the sparsity by using the double-threshold sigmoid penalty (DTSP) function. Then, the sparse reconstruction technique was used to estimate the 2-D angles. A three-parallel nested array structure was proposed in Ref. [27]. The three-parallel nested arrays with M elements can get DOF $O\left({\left(M/2\right)}^{2}\right)$. The continuity of virtual array elements was proved. 2-D angles were estimated by sparse reconstruction.

In recent years, compressed sensing technology has become a hot spot in many fields, and the problem of DOA estimation based on sparse reconstruction has attracted wide attention of researchers. All of Refs. [25,26,27] use sparse reconstruction technology. The disadvantage of this method is its high computational complexity when it is used in the angle estimation of sparse arrays [28,29,30,31,32]. A sparse array makes the original array aperture enlarged to increase the degree of freedom of angle estimation by vectorizing a sample covariance matrix, which forms a single measurement vector (SMV) model [33,34,35,36]. At the same time, the size of the sparse dictionary matrix is enlarged. The computational complexity of sparse reconstruction is directly related to the size of the dictionary [37,38,39,40,41]. This paper presents a solution to the problem of increasing computational complexity caused by the enlargement of sparse dictionary size in 2-D nested arrays.

The main content of this paper can be summarized as follows:

(1) Two-parallel nested arrays are proposed, which can separate the angles of the two dimensions. Two-parallel nested arrays can provide more continuous DOF than three-parallel nested subarrays proposed by Ref. [26].
(2) 2-D angle estimation models are constructed. Then, the data of subarray 1 are rearranged in reverse order, and the covariance matrix of rearranged subarray 1 data and subarray 2 data is obtained. After vectorizing the matrix, the 2-D angles are decoupled, and the single measurement vector (SMV) model is established.
(3) The SMV model of a virtual array is changed into multiple measurement vector (MMV) models to reduce the size of sparse dictionaries by a smoothing reconstruction method. By processing the SVD of the observation matrix, the algorithm further reduces the noise impact and computational complexity.
(4) A method based on sparse Bayesian learning is derived for MMV models. The angles of one dimension are estimated.
(5) The angles of the other dimension are estimated through the joint covariance matrix of the two subarrays. The estimated values from two dimensions can be automatically paired.

The two-parallel nested array proposed in this paper can be used in the field of passive reconnaissance and wireless networks.

The rest of this paper is organized as follows. The array model and signal model are introduced in Section 2. The 2-D DOA estimation algorithm for the proposed two-parallel nested arrays is described in detail in Section 3. Simulation results are presented to verify the performance of the proposed two-parallel nested arrays and the 2-D DOA estimation algorithm in Section 4. Conclusions are drawn in Section 5.

The following notations are used throughout the manuscript. We use bold lowercase letters to denote vectors (e.g., a), bold capitals for matrices (e.g., A) and hollow capitals for sets (e.g., A). For a matrix A, the symbols $A^{*}$, $A^{T}$ and $A^{H}$ denote the conjugation, transpose and conjugate transpose, respectively. ${\left[A\right]}_{i,:}$ denotes the vector consisting of the elements of the i-th row of the matrix A. ${\left[A\right]}_{:,j}$ denotes the vector consisting of the elements of the j-th column of the matrix A. ${\left[A\right]}_{i,j}$ denotes the j-th element of the i-th row of the matrix A. diag{a} denotes a diagonal matrix that uses the elements of vector a as its diagonal elements. vec{A} denotes vectorization, which converts the matrix A into a column vector by stacking the columns of A on top of one another. The symbol ⊙ denotes the Khatri-Rao product. ${∥\cdot ∥}_{F}$ denotes the Frobenius norm of a matrix.

## 2. Array Model and Signal Model

In this section, a two-parallel nested array is proposed, and the signal reception model of the proposed array is established.

**Figure 1:**
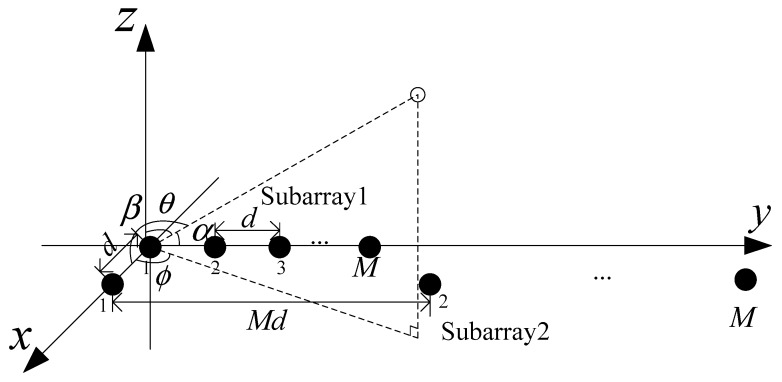
Two-parallel nested array structure.

Unlike traditional uniform parallel arrays, a two-parallel nested array model is proposed, as shown in Figure 1. Two-parallel nested arrays consist of two uniform linear arrays spaced at half of the signal wavelength λ. One of them is a compact array and the other is a sparse array. The two subarray elements have the same number of elements. In Figure 1, subarray 1 is a compact array composed of M elements, whose interval is $d=\frac{\lambda }{2}$. The number of elements in subarray 2 is M, and the interval between elements is Md. The parallel nested array is located on the x−y plane [24].

In this paper, K narrowband far-field source signals impinging on the parallel nested array with 2M elements are considered. The K source signals come from the $\left(\theta_{k},\phi_{k}\right)$, k=1,2,⋯,K direction where $\theta_{k}\in [0,\frac{\pi }{2})$ and $\phi_{k}\in [0,2\pi )$ denote the elevation and the azimuth angle of the k-th signal, respectively. $\left(\alpha_{k},\beta_{k}\right)$ is regarded as the direction of the k-th signal source, where $\cos\alpha_{k}=\sin\theta_{k}\sin\phi_{k}$, $\cos\beta_{k}=\sin\theta_{k}\cos\phi_{k}$. $\alpha_{k}$ denotes the angle between the k-th signal source and the y-axis, and $\beta_{k}$ denotes the angle between the k-th signal source and the x-axis. The two subarray outputs can be given as

(1) $$y_{1}(t)=\sum_{k=1}^{K}a_{1}(\alpha_{k})s_{k}(t)+n_{1}(t)=A_{1}(\alpha )s(t)+n_{1}(t),$$

(2) $$y_{2}(t)=\sum_{k=1}^{K}a_{2}(\alpha_{k})a(\beta_{k})s_{k}(t)+n_{2}(t)=A_{2}(\alpha )A(\beta )s(t)+n_{2}(t),$$

where $y_{1}(t)$, $y_{2}(t)$ denote the t-th snapshot vector of subarray 1 and subarray 2. $s(t)={\left[s_{1}(t),s_{2}(t),\cdots ,s_{K}(t)\right]}^{T}$ is the source signal vector. $n_{1}(t)$, $n_{2}(t)$ denote temporarily complex-valued white Gaussian noise with zero-mean and variance $\sigma_{n}^{2}$, respectively. $A_{1}(\alpha )=[a_{1}(\alpha_{1}),a_{1}(\alpha_{2}),\cdots ,a_{1}(\alpha_{K})]$, $A_{2}(\alpha )=[a_{2}(\alpha_{1}),a_{2}(\alpha_{2}),\cdots ,a_{2}(\alpha_{K})]$ are steering vector matrixes of subarray 1 and subarray 2, where

(3) $$a_{1}(\alpha_{k})={\left[1,e^{j2\pi d\cos(\alpha_{k})/\lambda },\cdots ,e^{j2\pi d(M-1)\cos(\alpha_{k})/\lambda }\right]}^{T},$$

(4) $$a_{2}(\alpha_{k})={\left[1,e^{j2\pi Md\cos(\alpha_{k})/\lambda },\cdots ,e^{j2\pi d(M-1)M\cos(\alpha_{k})/\lambda }\right]}^{T},$$

where $a(\beta_{k})=\exp[j2\pi Md/\cos(\beta_{k})]$ is the scalar containing the angles relative to the x-axis. A(β) can be expressed as

(5) $$A(\beta )=\mathrm{diag}\{a(\beta_{1}),a(\beta_{2}),\cdots ,a(\beta_{K})\}.$$

## 3. The Proposed 2-D DOA Estimation Algorithm for Parallel Nested Array

This section is divided into four parts. In the first part, a nested array angle estimation model is established, and the 2-D angle estimation is transformed into a 1-D angle estimation problem. In the second part, the complexity of the angle estimation model is reduced through the method proposed in this paper. In the third part, a sparse Bayesian learning algorithm is used to solve the problem of MMV angle estimation. In the fourth part, the angles of two dimensions are estimated, separately, and automatically paired.

### 3.1. Virtual Array Data Model

The data received by subarray 1 is rearranged in reverse order.

(6) $${\tilde{y}}_{1}(t)=J_{M}y_{1}(t)=J_{M}A_{1}(\alpha )s(t)+J_{M}n_{1}(t)={\tilde{A}}_{1}(\alpha )s(t)+{\tilde{n}}_{1}(t),$$

where $J_{M}$ is a M×M backward identity matrix. ${\tilde{A}}_{1}(\alpha )$ denotes the steering vector matrix in reverse order. ${\tilde{A}}_{1}(\alpha )=J_{M}A_{1}(\alpha )=[{\tilde{a}}_{1}(\alpha_{1}),{\tilde{a}}_{1}(\alpha_{2}),\cdots ,{\tilde{a}}_{1}(\alpha_{K})]$. The k-th column of ${\tilde{A}}_{1}(\alpha )$ is ${\tilde{a}}_{1}(\alpha_{k})={\left[e^{j2\pi d(M-1)\cos(\alpha_{k})/\lambda },e^{j2\pi d(M-2)\cos(\alpha_{k})/\lambda },\cdots ,1\right]}^{T}$. ${\tilde{n}}_{1}(t)$ is a noise vector in reverse order. The cross-covariance matrix of the two subarrays can be expressed as

(7) $$\begin{array}{ll} R & =E\{{\tilde{y}}_{1}(t)y_{2}^{H}(t)\}\\ & =E\{{\tilde{A}}_{1}(\alpha )s(t)s^{H}(t)A^{H}(\beta )A_{2}^{H}(\alpha )\}+E\{{\tilde{n}}_{1}(t)n_{2}^{H}(t)\}\\ & ={\tilde{A}}_{1}(\alpha )R_{s}A^{*}(\beta )A_{2}^{H}(\alpha ) \end{array},$$

where $R_{s}=\mathrm{diag}\{\rho_{1}^{},\rho_{2}^{},\cdots \rho_{K}^{}\}$ represents the covariance matrix of the K signals. $\rho_{k}^{}$ denotes signal power of the k-th signal. $A^{*}(\beta )=A^{H}(\beta )$, because A(β) is a diagonal matrix. The covariance of the noise is eliminated since the noise of the two subarrays is uncorrelated.

We define $\Omega =R_{s}A^{*}(\beta )=\mathrm{diag}\{\rho_{1}a(\beta_{1}),\rho_{2}a(\beta_{2}),\cdots ,\rho_{K}a(\beta_{K})\}$. In order to expand the degree of freedom, we calculate y by vectorizing cross-covariance matrix R.

(8) $$\begin{array}{ll} y & =\mathrm{vec}(R)=\mathrm{vec}({\tilde{A}}_{1}(\alpha )\Omega )\\ & =(A_{2}^{*}(\alpha )⊙{\tilde{A}}_{1}(\alpha ))p\\ & =A_{v}p \end{array},$$

where $p=[\rho_{1}a(\beta_{1}),\rho_{2}a(\beta_{2}),\cdots ,\rho_{K}a(\beta_{K})]$ is regarded as the input value of the virtual array. $A_{v}=A_{2}^{*}(\alpha )⊙{\tilde{A}}_{1}(\alpha )=[a_{2}^{*}(\alpha_{1})⊗{\tilde{a}}_{1}(\alpha_{1}),a_{2}^{*}(\alpha_{2})⊗{\tilde{a}}_{1}(\alpha_{2}),\cdots ,a_{2}^{*}(\alpha_{K})⊗{\tilde{a}}_{1}(\alpha_{K})]\in {\mathbb{C}}^{MM\times K}$ is viewed as the steering vector matrix for the virtual array.

(9) $$a_{v}(\alpha_{k})={\left[e^{-j(M-1)2\pi d\cos\alpha_{k}/\lambda },e^{-j(M-2)2\pi d\cos\alpha_{k}/\lambda },\cdots ,e^{j(M-1)M2\pi d\cos\alpha_{k}/\lambda }\right]}^{T}\in {\mathbb{C}}^{M^{2}\times 1}$$

The virtual array’s degree of freedom is $M^{2}$. Unlike the virtual array corresponding to a 1-D nested array [20], there is no duplicate term in the output of the virtual array of the parallel nested array.

According to the Equation (8), the angles of two dimensions, $\alpha_{k}$ and $\beta_{k}$ are separated into two items, $A_{v}$ and p, respectively. In order to estimate the value of the angle $\alpha_{k}$, k=1,2,⋯,K, a sparse SMV model is established as follows.

(10) $$y=\Phi \tilde{p}+e,$$

where Φ is a sparse dictionary matrix in compressed sensing. $\left\{\varphi_{1},\varphi_{2},\cdots ,\varphi_{N}\right\}$ is a fixed sampling grid set covering all possible $\Phi =[a(\varphi_{1}),a(\varphi_{2}),\cdots ,a(\varphi_{N})]\in {\mathbb{C}}^{MM\times N}$. $\tilde{p}$ is a sparse representation of input values of the virtual array. In $\tilde{p}$, only K items are nonzero, and the rest are zeros. e is a data error vector since the number of arrays sampling is finite, resulting in the error between the real value of R and the measured value.

### 3.2. Reducing the Size of the Sparse Dictionary

**Figure 2:**
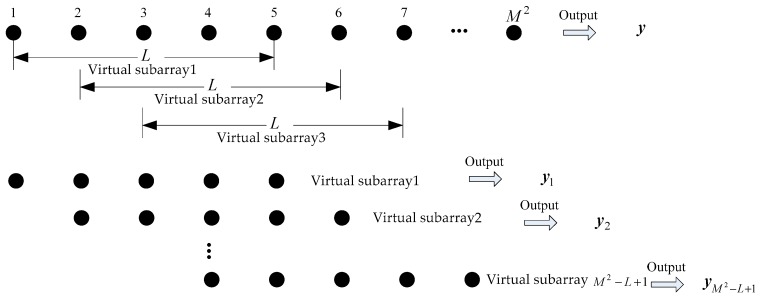
Schematic diagram of virtual subarrays.

The number of elements in the virtual array is $M^{2}$, so the size of the dictionary matrix is $M^{2}\times N$, where N denotes the number of sparse divisions in airspace. The size of the dictionary matrix increases squared with the array elements of nested array subarrays. More importantly, the size of the dictionary determines the computational complexity of the sparse algorithm. It can effectively reduce the computational complexity of the algorithm to reduce the size of the dictionary. As the output of virtual array, $y={\left[y_{1},y_{2},\cdots ,y_{M^{2}}\right]}^{T}$ is smoothly divided into overlapping virtual subarrays with L $\left(K\leq L\leq M^{2}\right)$ elements in order to reduce the size of the dictionary. The number of virtual subarrays is $M^{2}-L+1$, as shown in Figure 2. The output value of the i-th virtual subarray is $y_{i}={\left[y_{i},y_{i+1},\cdots ,y_{i+L-1}\right]}^{T}$, $i=1,2,\cdots ,M^{2}-L+1$. A new observation matrix Y is constructed with the output of each virtual subarray as a column, namely $Y=[y_{1},y_{2},\cdots ,y_{M^{2}-L+1}]$. The output sparse model of the virtual array can be redefined as a MMV model, as shown below,

(11) $$Y=\tilde{\Phi }P+E,$$

where $\tilde{\Phi }=[\tilde{a}(\varphi_{1}),\tilde{a}(\varphi_{2}),\cdots ,\tilde{a}(\varphi_{N})]\in {\mathbb{C}}^{L\times N}$. $\tilde{a}(\varphi_{1})$ denotes the steering vector of virtual subarrays. Obviously, the size of $\tilde{\Phi }$ is much smaller than that of Φ. $P\in {\mathbb{C}}^{N\times (M^{2}-L+1)}$ is the sparse input matrix of virtual subarrays. There are K (K≪N) nonzero rows in P, and the rest are 0. E denotes a sparse representation of the noise term. Indexed set K of nonzero rows in P can be represented as

(12) $$K=\{k\in N|P_{k,:}\neq 0\}.$$

When the set K is known, the estimated value of $\alpha_{i}$ (i=1,2,⋯,K) can be obtained by matrix $\tilde{\Phi }$.

To eliminate data noise, the SVD is applied to matrix Y. Y can be expressed as $Y=U\Gamma V^{T}$ by SVD, where U and V denote left and right singular matrices of Y, respectively. In this paper, we assume that the value of the number of signal sources K is known. Γ is a diagonal matrix consisting of singular values of Y. The first K values on the diagonal line in Γ correspond to the signal subspace in Y, and the rest of the values correspond to noise subspace. Equation (11) can be rewritten as follows,

(13) $$Y_{s}=\tilde{\Phi }P_{s}+E_{s},$$

where $Y_{s}=YVD$, $P_{s}=\mathrm{PVD}$, $E_{s}=\mathrm{EVD}$, $D=\left[\begin{array}{cc} I_{K} & 0\\ 0 & 0 \end{array}\right]$. Through SVD, the size of the observation matrix Y is further reduced, and some of the noise in the observation data is removed, which is conducive to improving the signal-to-noise ratio (SNR).

### 3.3. Sparse Bayesian Learning Algorithm for MMV

Sparse Bayesian learning is applied to solve the MMV problem in Equation (13). We assume that noise items $E_{s}$ in Equation (13) obey complex-value Gaussian distribution with zero mean and variance $\sigma_{}^{2}$. The posterior probability density of the observed matrix $Y_{s}$ is

(14) $$\mathrm{Pr}(Y_{s}|P_{s};\sigma^{2})=\frac{1}{{\left(\pi \sigma^{2}\right)}^{L(M^{2}-L+1)}}\exp(-\frac{{∥Y_{s}-\tilde{\Phi }P_{s}∥}_{F}^{2}}{\sigma^{2}}).$$

Suppose the variable $P_{s}$ obeys zero-mean complex-value Gaussian distribution. The variance of $P_{s}$ is Λ=diag(γ), where $\gamma =[\gamma_{1},\gamma_{2},\cdots ,\gamma_{N}{]}^{T}$ is a hyperparameter that needs to be estimated. $\gamma_{i}$ (i=1,2,⋯,N) is a scalar, which represents the variance of the i-th row in $P_{s}$. The i-th row in $P_{s}$ is a nonzero vector if $\gamma_{i}>0$.

(15) $$\mathrm{Pr}(P_{s};\gamma )=\prod_{n=1}^{N}CN(0,\Lambda )$$

Equation (12) can be rewritten as follows,

(16) $$K=\{k\in N|\gamma_{k}>0\}.$$

According to the Bayesian principle, the conditional probability density of $P_{s}$ is

(17) $$\mathrm{Pr}(P_{s}|Y_{s};\gamma ,\sigma^{2})=\frac{\mathrm{Pr}(Y_{s}|P_{s};\sigma^{2})\mathrm{Pr}(P_{s};\gamma )}{\mathrm{Pr}(Y_{s};\gamma ,\sigma^{2})}.$$

Ignoring the normalized denominator, we get

(18) $$\begin{array}{ll} \mathrm{Pr}(P_{s}|Y_{s};\gamma ,\sigma^{2}) & \propto \mathrm{Pr}(Y_{s}|P_{s};\sigma^{2})\mathrm{Pr}(P_{s};\gamma )\\ & \propto \frac{\exp\left\{-\mathrm{tr}\left[{\left(P_{s}-\mu_{P_{s}}\right)}^{H}\Sigma_{P}^{-1}(P_{s}-\mu_{P_{s}})\right]\right\}}{{\left(\pi^{L}\det(\Sigma_{P_{s}}^{})\right)}^{M^{2}-L+1}}\\ & =CN(\mu_{P_{s}},\Sigma_{P_{s}}^{}) \end{array}$$

where $\mu_{P_{s}}$ is the mean of $P_{s}$, and $\Sigma_{P_{s}}^{}$ is the variance of $P_{s}$. According to Equations (14) and (15), $\mu_{P_{s}}$ and $\Sigma_{P_{s}}$ can be estimated by the following formula,

(19) $$\mu_{P_{s}}=\Lambda {\tilde{\Phi }}_{}{}^{H}\Sigma_{Y_{s}}^{-1}Y_{s},$$

(20) $$\Sigma_{P_{s}}={\left(\frac{1}{\sigma^{2}}{\tilde{\Phi }}^{H}\tilde{\Phi }+\Lambda^{-1}\right)}^{-1}=\Lambda -\Lambda {\tilde{\Phi }}^{H}\Sigma_{Y_{s}}^{-1}\tilde{\Phi }\Lambda ,$$

where

(21) $$\Sigma_{Y_{s}}=E(Y_{s}{}^{H}Y_{s})=\sigma^{2}I_{L}+\tilde{\Phi }\Lambda {\tilde{\Phi }}^{H}.$$

Using the inverse lemma of matrix, $\Sigma_{Y_{s}}^{-1}$ can be obtained:

(22) $$\begin{array}{ll} \Sigma_{Y_{s}}^{-1} & =\sigma^{-2}I_{L}-\sigma^{-2}\tilde{\Phi }{\left(\frac{1}{\sigma^{2}}{\tilde{\Phi }}_{}^{H}{\tilde{\Phi }}_{}+\Lambda^{-1}\right)}^{-1}{\tilde{\Phi }}^{H}\sigma^{-2}\\ & =\sigma^{-2}I_{L}-\sigma^{-2}\tilde{\Phi }\Sigma_{P_{s}}{\tilde{\Phi }}^{H}\sigma^{-2} \end{array}.$$

The denominator of Equation (17) can be obtained

(23) $$\mathrm{Pr}(Y_{s};\gamma ,\sigma^{2})=\int \mathrm{Pr}(Y_{s}|P_{s};\sigma^{2})\mathrm{Pr}(P_{s};\gamma )dP_{s}=\frac{\exp\left\{-\mathrm{tr}(Y_{s}{}^{H}\Sigma_{Y_{s}}^{-1}Y_{s})\right\}}{{\left(\pi^{L}\det\Sigma_{Y_{s}}\right)}^{M^{2}-L+1}}.$$

The hyperparameters γ and $\sigma^{2}$ can be estimated by

(24) $$(\hat{\gamma },{\hat{\sigma }}^{2})=\underset{\gamma \geq 0,\sigma^{2}>0}{\arg\max}\log\mathrm{Pr}(Y_{s};\gamma ,\sigma^{2}),$$

where

(25) $$\begin{array}{ll} \log\mathrm{Pr}(Y_{s};\gamma ,\sigma^{2}) & \propto -\mathrm{tr}(Y_{s}{}^{H}\Sigma_{Y_{s}}^{-1}Y_{s})-(M^{2}-L+1)\log\det\Sigma_{Y_{s}}\\ & \propto -\mathrm{tr}(\Sigma_{Y_{s}}^{-1}R_{Y_{s}})-\log\det\Sigma_{Y_{s}} \end{array}.$$

In Equation (25), $R_{Y_{s}}=Y_{s}Y_{s}{}^{H}/(M^{2}-L+1)$. We can obtain

(26) $$\frac{\partial \Sigma_{Y_{s}}^{-1}}{\partial \gamma_{m}}=-\Sigma_{Y_{s}}^{-1}\frac{\partial \Sigma_{Y_{s}}}{\partial \gamma_{m}}\Sigma_{Y_{s}}^{-1}=-\Sigma_{Y_{s}}^{-1}\tilde{a}(\varphi_{m}){\tilde{a}}^{H}(\varphi_{m})\Sigma_{Y_{s}}^{-1},$$

(27) $$\frac{\partial \log\det\Sigma_{Y_{s}}}{\partial \gamma_{m}}=\mathrm{tr}\left(\Sigma_{Y_{s}}^{-1}\frac{\partial \Sigma_{Y_{s}}}{\partial \gamma_{m}}\right)={\tilde{a}}^{H}(\varphi_{m})\Sigma_{Y_{s}}^{-1}\tilde{a}(\varphi_{m}).$$

The derivatives of Equation (25) can be expressed as

(28) $$\frac{\partial \log\mathrm{Pr}(Y_{s};\gamma ,\sigma^{2})}{\partial \gamma_{m}}=\frac{1}{\gamma_{m}^{2}(M^{2}-L+1)}{∥\mu_{m}∥}_{2}^{2}-{\tilde{a}}^{H}(\varphi_{m})\Sigma_{Y_{s}}^{-1}\tilde{a}(\varphi_{m}),$$

where $\mu_{m}=\gamma_{m}{\tilde{a}}^{H}(\varphi_{m})\Sigma_{Y_{s}}^{-1}Y_{s}$, $\mu_{m}$ is the m-th row of $\mu_{P}$. In order to calculate Equation (28), the iterative formula of $\gamma_{m}$ is

(29) $$\gamma_{m}^{new}=\frac{1}{\sqrt{M^{2}-L+1}}{∥\mu_{m}∥}_{2}/\sqrt{{\tilde{a}}^{H}(\varphi_{m})\Sigma_{Y_{S}}^{-1}\tilde{a}(\varphi_{m})}.$$

According to Equation (21), the covariance of $Y_{s}$ can also be expressed as

(30) $$\Sigma_{Y_{S}}=\sigma^{2}I+{\left[\tilde{\Phi }\right]}_{:,K}\Lambda_{K}{\left[{\tilde{\Phi }}^{H}\right]}_{K,:},$$

where ${\left[\tilde{\Phi }\right]}_{:,K}$ denotes a matrix, which is composed of columns of $\tilde{\Phi }$ corresponding to set K in matrix $\tilde{\Phi }$. $\Lambda_{K}$ represents a diagonal matrix consisting of elements in Λ corresponding to K. When the optimal solution of $\mu_{P_{s}}$ and $\Sigma_{Y_{s}}$ is obtained, Equations (21) and (30) should be the same. The following equality is obtained,

(31) $${\left[{\tilde{\Phi }}^{H}\right]}_{K,:}(R_{Y_{s}}-\Sigma_{Y_{s}}){\left[\tilde{\Phi }\right]}_{:,K}=0.$$

Substituting Equation (30) into Equation (31), we can obtain

(32) $${\left[{\tilde{\Phi }}^{H}\right]}_{K,:}(R_{Y_{s}}-\sigma^{2}I){\left[\tilde{\Phi }\right]}_{:,K}={\left[{\tilde{\Phi }}^{H}\right]}_{K,:}{\left[\tilde{\Phi }\right]}_{:,K}\Lambda_{K}{\left[{\tilde{\Phi }}^{H}\right]}_{K,:}{\left[\tilde{\Phi }\right]}_{:,K}.$$

The iterative formula of $\sigma^{2}$ can be expressed as

(33) $${\left(\sigma^{2}\right)}^{\mathrm{new}}=\frac{1}{L-K}\mathrm{tr}[(I-{\left[\tilde{\Phi }\right]}_{:,K}{\left[{\tilde{\Phi }}^{H}\right]}_{K,:}^{†})R_{Y_{s}}],$$

where ${\left[{\tilde{\Phi }}^{H}\right]}_{K,:}^{†}={\left({\left[{\tilde{\Phi }}^{H}\right]}_{K,:}{\left[\tilde{\Phi }\right]}_{:,K}\right)}^{-1}{\left[{\tilde{\Phi }}^{H}\right]}_{K,:}$ is the Moore-Penrose pseudo-inverse operation of ${\left[{\tilde{\Phi }}^{H}\right]}_{K,:}$. Hyper parameters $\gamma ,\sigma^{2}$ can be estimated by Equations (29) and (33), and K can be obtained. $\alpha_{i}$ (i=1,2,⋯,K) can be estimated.

### 3.4. The Method to Estimate $\beta_{i}$

The method of estimating $\beta_{i}$ (i=1,2,⋯,K) is presented in this section under the condition of known estimated value ${\hat{\alpha }}_{i}$. According to Equation (7), the cross-covariance matrix of the input of subarray 1 and subarray 2 can be expressed as

(34) $$R={\tilde{A}}_{1}(\hat{\alpha })R_{s}A^{*}(\beta )A_{2}^{H}(\hat{\alpha }),$$

where ${\tilde{A}}_{1}(\hat{\alpha })$ denotes the steering vector matrix of subarray 1 expressed with known estimations ${\hat{\alpha }}_{i}$ (i=1,2,⋯,K). $A_{2}^{H}(\hat{\alpha })$ represents part of the steering vector matrix of subarray 2, and the other part $A^{*}(\beta )$ is unknown. The estimated value of $A^{*}(\beta )$ can be expressed as a form of minimum mean square error.

(35) $$A(\beta )=\arg\underset{A(\beta )}{\min}{∥R-{\tilde{A}}_{1}(\hat{\alpha })R_{s}A^{*}(\beta )A_{2}^{H}(\hat{\alpha })∥}_{F}^{2},$$

where $R=\frac{1}{T}\sum_{t=1}^{T}J_{M}y_{1}(t)y_{2}^{H}(t)$ is known. The autocorrelation matrix $R_{s}$ of signals s(t) is unknown. In order to estimate $R_{s}$, eigenvalue decomposition is applied to R.

(36) $$R=U_{r}\Gamma_{r}U_{r}^{H},$$

where $U_{r}=[U_{rs},U_{rn}]$. $U_{rs}$ denotes a matrix composed of eigenvectors corresponding to signal subspaces. $U_{rn}$ denotes the eigenvector matrix corresponding to noise subspaces. $\Gamma_{r}$ is a diagonal matrix composed of eigenvalues in descending order.

(37) $$R=U_{rs}\Gamma_{rs}U_{rs}^{H}+U_{rn}\Gamma_{rn}U_{rn}^{H}\;$$

$\Gamma_{rs}$ and $\Gamma_{rn}$ denote diagonal matrixes composed of signal eigenvalues and noise eigenvalues, respectively. The autocorrelation matrix $R_{1}$ of subarray 1 can be expressed as

(38) $$R_{1}=E\{y_{1}(t)y_{1}^{H}(t)\}=A_{1}(\hat{\alpha })R_{s}A_{1}^{H}(\hat{\alpha })+\sigma_{n}^{2}I.$$

According to Equations (37) and (38), the columns of $A_{1}(\hat{\alpha })$ and $U_{rs}$ span the same space. $R_{s}$ can be estimated as follows.

(39) $$R_{s}=A_{1}^{+}(\hat{\alpha })U_{rs}\Gamma_{rs}U_{rs}^{H}{\left(A_{1}^{H}(\hat{\alpha })\right)}^{+}\;$$

A(β) can be estimated by

(40) $$\hat{A}(\beta )={\left({\hat{R}}_{s}^{-1}{\tilde{A}}_{1}^{+}(\hat{\alpha })R{\left(A_{2}^{H}(\hat{\alpha })\right)}^{+}\right)}^{*}.$$

The closed-form expression of $\hat{A}(\beta )$ estimation can be obtained by

(41) $$\hat{A}(\beta )={\left({\left(A_{1}^{+}(\hat{\alpha })U_{rs}\Gamma_{rs}U_{rs}^{H}{\left(A_{1}^{H}(\hat{\alpha })\right)}^{+}\right)}^{-1}{\tilde{A}}_{1}^{+}(\hat{\alpha })R{\left(A_{2}^{H}(\hat{\alpha })\right)}^{+}\right)}^{*}.$$

$\beta_{i}$(i=1,2,⋯,K) can be estimated by

(42) $${\hat{\beta }}_{i}=\underset{\beta_{i}}{\arg}(\hat{A}(\beta_{i})).$$

The estimated values of $\alpha_{i}$ and $\beta_{i}$ can be automatically matched.

The computational complexity of the proposed two-dimensional angle estimation is $O\{M^{2}N+8MK^{2}+4M(4M-K)K+8(M-1)K^{2}\}$.

## 4. Simulation

In this section, several simulation experiments are provided to verify the superiority of the two-parallel nested arrays and the proposed estimation algorithm. This section is divided into three simulations. In the first simulation, the degree of freedom of two-parallel nested arrays is verified. In the second simulation, the 2-D DOA estimation algorithm proposed in this paper is tested and verified. In the third simulation, the proposed method is compared with the other three methods in the literature. The simulation software I use is MATLAB R2014a. My personal computer’s CPU is Intel Core i7.

### 4.1. Comparison of DOF in Parameter Estimation

**Figure 3:**
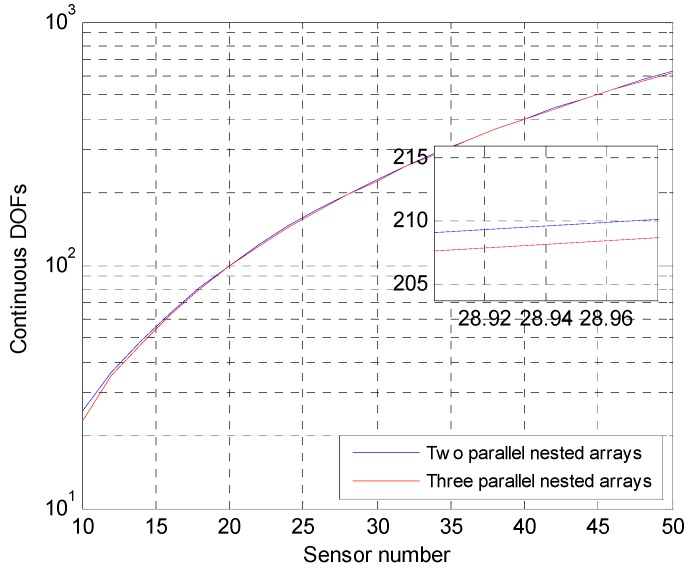
DOF comparison.

The DOF of the two-parallel nested arrays proposed in this paper is compared with that of the three-parallel nested arrays [26]. In Ref. [26], three-parallel nested arrays can offer $M^{2}-1$ DOF with 2M sensors, while the two-parallel nested arrays proposed in this paper can offer $M^{2}$ DOF with 2M sensors. In Figure 3, when the elements of the two arrays are from 10 to 50, the continuous degree of freedom changes of the array are counted. The blue line represents the continuous DOF of two-parallel nested arrays proposed in this paper. The red line represents a continuous DOF of three-parallel nested arrays proposed in Ref. [26].

As can be seen from the Figure 3, the continuous DOF of the two-parallel nested arrays is slightly larger than that of the three-parallel nested arrays. This shows that the two-parallel nested arrays proposed in this paper have better array structure than Ref. [26]. In practical applications, we can estimate a greater number of radiation source angle values. Moreover, the array structure proposed in this paper is simpler than the structure of Ref. [26].

### 4.2. Results of 2-D DOA Estimation

In this part, the DOA estimation performance of the proposed algorithm for 2-D angle estimation is verified. The number of sensors in subarray 1 and subarray 2 is M=5. The directions of the 10 far-field narrowband signal sources are $\left(\alpha_{i},\beta_{i}\right)\in \{\left(10,53\right),\left(15,25\right),\left(20,45\right),\left(25,55\right),\left(30,16\right),\left(35,20\right),\left(45,37\right),\left(55,55\right),\left(60,12\right),\left(70,33\right)\}$ i=1,2,⋯,10. The signal is received by the two-parallel array and estimated by the 2-D direction-finding algorithm proposed in this paper with the signal-to-noise ratio SNR=10dB and the number of snapshots P=500. The initialization parameters of the DOA estimation algorithm are $\sigma_{0}^{2}=0.5$, ε=0.1, $\gamma_{0}=0.1$, K=10. The angle interval in the sparse dictionary is $0.1^{\circ }$.

**Figure 4:**
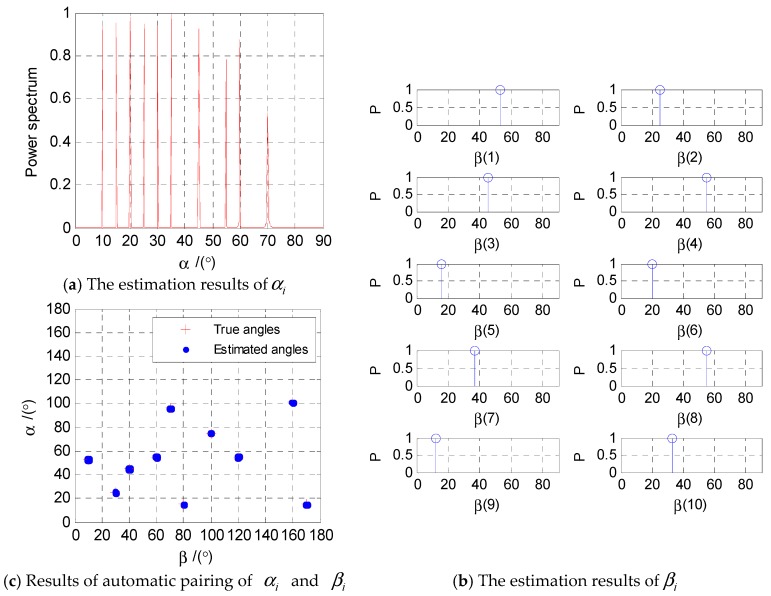
2-D DOA estimation results.

As shown in Figure 4a, the angle $\alpha_{i}$ (i=1,2,⋯,10) is accurately estimated by the multiple measurement vector based on the sparse Bayesian learning (MMV-SBL) direction finding algorithm proposed in this paper. It can be seen from the Figure 4a that the proposed MMV-SBL algorithm can estimate the angle $\alpha_{i}$ (i=1,2,⋯,10). The 10 peaks of the estimation result are obvious and sharp, and there is no false sidelobe.

Figure 4b shows the estimation result of angle $\beta_{i}$(i=1,2,⋯,10) through the Equations (41) and (42) at the known angle $\alpha_{i}$. The angle order in Figure 4b is in one-to-one correspondence with Figure 4a. As can be seen from Figure 4b, angle $\beta_{i}$ (i=1,2,⋯,10) can be estimated by the algorithm proposed in this paper (Algorithm 1), and the angles of the two dimensions are automatically paired.

Figure 4c shows the paired results of two-dimensional angles $\alpha_{i}$ and $\beta_{i}$ with 50 Monte Carlo experiments. The red crosses represent the true values of the 2-D angles, and the blue dots represent the estimated angles. As shown in Figure 4c, the blue dots are concentrated near the red crosses. Obviously, the 2-D DOA estimation algorithm presented in this paper shows excellent estimation results.

**Algorithm 1.** The proposed algorithm steps for the 2-D angle estimation of parallel nested array.**Input:** Data vectors obtained by subarray 1 and subarray 2, $y_{1}(t)$ and $y_{2}(t)$. Step 1. By Equation (6), calculate ${\tilde{y}}_{1}(t)$. By Equations (7) and (8), calculate R and vectorize R.Step 2. Smooth reconstruct y and get Y. Use SVD to Y and get $Y_{s}$.Step 3. Initialize parameters $\sigma_{0}^{2}$, ε, $\gamma_{0}$, j=1, K.Step 4. $\sigma_{j}^{2}=\sigma_{j-1}^{2}$, $\gamma_{j}=\gamma_{j-1}$, $\Lambda =diag(\gamma_{j})$. $\Sigma_{Y_{s}}$ and $\mu_{P_{s}}$ are calculated by Equations (19) and (21), separately.Step 5. By Equation (29), $\gamma_{m}$ is calculated. The index of K maximum values of vector $\gamma_{j}$ constitute the set K.Step 6. According to K, ${\left[\tilde{\Phi }\right]}_{:,K}$ is obtained. $\sigma_{j}^{2}$ is calculated by Equation (33)Step 7. If ${∥\gamma_{j}-\gamma_{j-1}∥}_{1}/{∥\gamma_{j-1}∥}_{1}<\varepsilon$, $\alpha_{i}$ is obtained by ${\left[\tilde{\Phi }\right]}_{:,K}$. If ${∥\gamma_{j}-\gamma_{j-1}∥}_{1}/{∥\gamma_{j-1}∥}_{1}\geq \varepsilon$, let j=j+1, and execute step 4.Step 8. By Equations (41) and (42), $\beta_{i}$ is obtained.**Output:** 2-D angle estimation $\alpha_{i}$ and $\beta_{i}$
(i=1,2,⋯,K).

### 4.3. Performance Comparison of Angle Estimation

In this subsection, TPCA [25], DTSP [26] and DETPA [24] with the respective 2-D DOA estimation methods are used as contrast objects. They all take the two-parallel array as the research object, but the array structure and direction-finding algorithm are different. Under different conditions, the array structure and algorithm proposed in this paper will be compared with these three methods. In this simulation, the sensor number of subarray 1 and subarray 2 in the proposed two-parallel array is $M_{1}=M_{2}=6$. The sensor numbers of two subarrays in Ref. [25] are $M_{1}=5$ and $M_{2}=7$. Those of two subarrays in Ref. [26] are $M_{1}=6$ and $M_{2}=5$. Those of two subarrays in Ref. [24] are $M_{1}=5$ and $M_{2}=7$. The number and angle information of the sources are the same as that in the simulation 2. We conducted 500 Monte Carlo experiments with the root-mean-square error (RMSE) as the evaluation criterion. The RMSE is defined as

(43) $$\mathrm{RMSE}=\sqrt{\frac{1}{QK}\sum_{q=1}^{Q}\sum_{i=1}^{K}\left({\left({\hat{\alpha }}_{i,q}-\alpha_{i}\right)}^{2}+{\left({\hat{\beta }}_{i,q}-\beta_{i}\right)}^{2}\right)},$$

where Q denotes the number of Monte Carlo trials, and ${\hat{\alpha }}_{i,q}$ denotes the i-th angle estimation in the q-th Monte Carlo trial.

**Figure 5:**
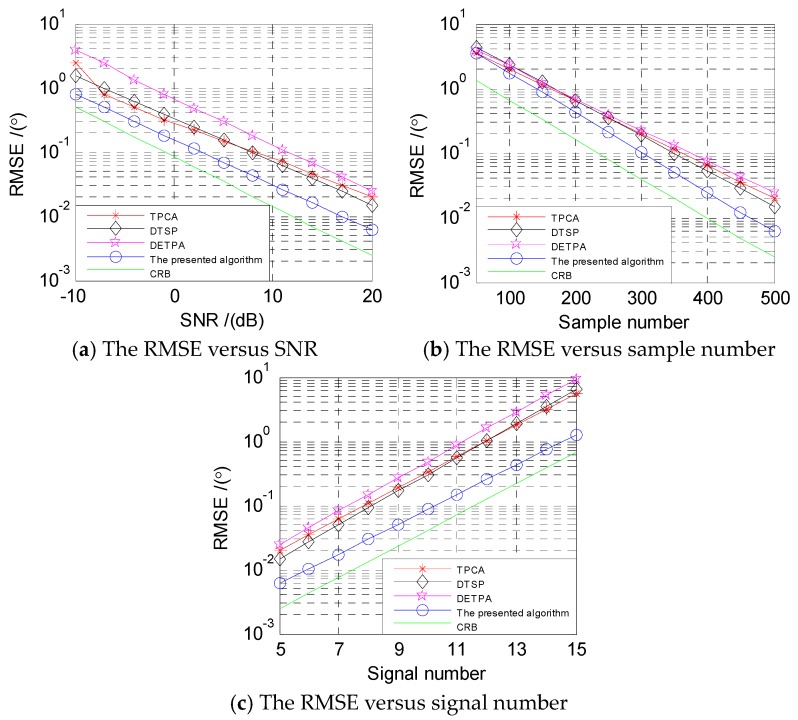
The algorithms’ performance comparison.

In Figure 5a, the RMSEs of four different array structures with their DOA estimation algorithms are calculated under different SNRs, from −10 dB to 20 dB, and the number of snapshots P=500. As shown in Figure 5a, under the same SNR condition, the DOA estimation performance of the algorithm proposed in this paper is superior to the other three algorithms.

In Figure 5b, SNR is fixed at 20 dB. The RMSEs of the proposed algorithm and the other three algorithms are counted with snapshots from 50 to 500. As shown in the figure, the RMSE of the proposed method is smaller than the other three methods, indicating that the former has the best DOA estimation performance.

In Figure 5c, the SNR is fixed at 20 dB and the number of snapshots is 500. The number of signals ranges from 5 to 15. The RMSEs of the proposed algorithm and other three algorithms are counted. The figure shows that the RMSE of the proposed algorithm is smaller than that of the other three algorithms when the number of the parallel array sensors is the same, and the performance advantage of the proposed algorithm is more obvious than that of the other three algorithms as the number of sensors increases.

All of the above are based on the simulation analysis under ideal conditions. In practical application, the signal received by the array may be a nonstationary signal. The noise may be Gaussian colored noise. The mutual coupling effect may exist between the elements of subarray 1, and the inconsistency of each channel of the elements may exist. All these will seriously affect the accuracy of DOA estimation. The corresponding models should be constructed to solve these practical problems. This is a problem that needs further study.

## 5. Conclusions

In this paper, a two-parallel nested array is proposed, which can provide $M^{2}$ degrees of freedom with 2M sensors. In order to estimate the 2-D angles of the signal sources, a 2-D DOA angle estimation algorithm based on sparse Bayesian learning is proposed. In this algorithm, the SMV model of the previous nested array is transformed into the MMV model to reduce the size of the sparse dictionary. Through SVD processing, the algorithm reduces the noise effect and computational complexity. We derive a sparse Bayesian learning algorithm for the MMV model. The simulation results show that the proposed two-parallel nested arrays have large DOF of parameter estimation, and the proposed DOA estimation algorithm also has excellent DOA estimation performance. In practical applications, the small interval between subarray 1 elements may result in a mutual coupling effect and the decrease of angle estimation accuracy. We find that the sparsity of the array can be increased and the degree of freedom can be improved by adjusting the position of subarray 1 elements, which leads to better direction-finding performance. We will further study how to adjust the element location of subarray 1 to achieve the optimal array structure.
